# Microstructure and Properties of Conventional Cast Versus Annular Laser-Clad Babbitt Alloy Layers for Sliding Bearings

**DOI:** 10.3390/mi17010134

**Published:** 2026-01-21

**Authors:** Jing Jin, Jun Ye, Hao Xue, Yongli Xu, Zhongwai Guo, Zhenghong Zhou, Gaohuan Xu, Guobiao Wang

**Affiliations:** 1 School of Mechanical Engineering, Tianjin University, Tianjin 300072, China; jinjing@zjvtit.edu.cn (J.J.); 20150009@zjipc.edu.cn (J.Y.); 2 Department of Rail Transit, Zhejiang Institute of Communications, Hangzhou 311112, China; 3 School of Electromechanical Engineering, Zhejiang Industry Polytechnic College, Shaoxing 312000, China; 4 Key Laboratory of Advanced Manufacturing Technology of Jiaxing City, College of Mechanical Engineering, Jiaxing University, Jiaxing 341001, China; xuehao@zjxu.edu.cn; 5 Zhejiang Shenfa Bearing Co., Ltd., Shaoxing 311800, China; yongli.xu@shenfagroup.com (Y.X.); gzwai@163.com (Z.G.); 15068987158@163.com (Z.Z.); 6 Geely Automotive Institute, Hangzhou Polytechnic University, Hangzhou 310018, China

**Keywords:** laser cladding, casting, babbitt alloy, annular laser cladding

## Abstract

Sliding bearing alloy layers must combine excellent tribological performance with reliable metallurgical bonding, but conventional fabrication methods often suffer from coarse grains, chemical segregation and poor interface adhesion. Annular coaxial laser wire-feed cladding, by providing more uniform heat input and rapid solidification, is expected to mitigate these deficiencies; however, systematic studies of this technique applied to tin-based Babbitt alloy layers remain limited. In this work, Babbitt layers produced by conventional casting and by annular coaxial laser wire-feed cladding were compared in terms of microstructure, phase constitution, hardness and tribological behavior. The results indicate that laser cladding can produce continuous, dense and well-bonded coatings and markedly refine the SnSb phase, reducing grain size from approximately 100 μm in the cast material to 10-20 μm. Hardness increased from 25.3 HB to 27.6 HB, while tribological performance improved substantially: the coefficient of friction decreased by about 38.19% and the wear volume was reduced by approximately 10.46%. These improvements are attributed mainly to the rapid solidification, low dilution and more uniform phase distribution associated with annular coaxial laser cladding, demonstrating the strong potential of this process for fabricating high-performance tin-based Babbitt bearing layers.

## 1. Introduction

Sliding bearings, as critical components of mechanical systems [1], exhibit exceptional multidirectional load-carrying capacity and dynamic stability [2]. These superior properties render them indispensable support elements for extreme-condition equipment such as nuclear reactor coolant pumps [3] and marine propulsion systems [4] while simultaneously serving pivotal roles in high-speed rotor systems including wind turbine generators [5,6], aerospace applications [7], high-speed rail transport [8] and mining machinery [9] under heavy impact loading conditions. The tribological properties of bearing lining materials directly determine the operational reliability and service life of machinery. Among these materials, tin-based Babbitt alloys have emerged as an ideal choice for sliding bearing alloy layers due to their superior anti-friction characteristics, seizure resistance, and wear durability [10]. Conventional Babbitt alloy fabrication techniques (e.g., casting, spraying, and brazing) often suffer from non-uniform cooling, coarse grains, compositional segregation, weak interfacial bonding, and low material utilization efficiency. In contrast, laser cladding technology, as an advanced additive manufacturing approach, enables precise control over the geometric parameters of alloy layers through accurate process regulation. This technique demonstrates remarkable advantages including refined microstructure, homogeneous composition, strong interfacial bonding, low dilution rate, and high material utilization efficiency [11,12,13,14,15], thereby providing an innovative solution for preparing high-performance bearing tin-based Babbitt alloy layers.

The single-beam Gaussian laser serves as the conventional heat source for laser cladding of Babbitt alloy layers. Kim et al. [16] systematically investigated laser-clad tin-based Babbitt alloys, confirming the viability of this technique as an effective alternative manufacturing approach. Li et al. [17] employed Gaussian laser cladding to produce Sn-Cu-Sb coatings metallurgically bonded to stainless steel, consisting of a Sn-matrix with dispersed Cu_6_Sn_5_ and SnSb intermetallic phases. Hao et al. [18] conducted a systematic study on the laser cladding process for tin-based Babbitt alloy coatings. The results revealed that the laser-clad alloy exhibited a uniform and fine-grained microstructure with metallurgical bonding to the substrate, demonstrating significantly higher interfacial strength than conventional cast alloys. In recent years, the novel luminous coaxial annular laser cladding technology has been introduced into the cladding field [19]. With its unique annular spot energy distribution, this innovative technique achieves more uniform heat input and more stable molten pool dynamics, effectively overcoming the inherent limitations of conventional Gaussian laser cladding such as edge heat accumulation and central overheating. Edvard et al. developed a novel powder cladding head based on annular laser beam technology, which enables axial powder feeding and adjustable laser beam intensity distribution (LBID) on workpiece surfaces. Through laser direct wire deposition on 304 stainless steel substrates, they successfully deposited Ni wires and observed that the dilution rate decreased with increasing workpiece illumination proportion (WIP) [20]. Subsequent research on 316L stainless steel powder cladding on 304 stainless steel substrates investigated the influence of LBID variation in axial powder feeding processes [21]. Alexander Kuznetsov [22] systematically investigated the cladding of AISI 316L powder onto SS 304 substrates, evaluating both process feasibility and the influence of processing parameters on powder catchment efficiency. Through systematic characterization of conventional parameters and WIP, Kotar [23] revealed their distinct impacts on process stability and clad geometry in coaxial wire-fed annular laser cladding of AISI 316L, with laser power demonstrating nonlinear dependence on workpiece irradiation proportion. Wang et al. [24] conducted simulation studies on the powder flow behavior in a novel internal-laser coaxial two-airflow powder feeding system. Subsequent annular laser direct metal deposition experiments demonstrated crack-free deposited layers without structural defects.

Although significant progress has been made in research on Gaussian laser cladding of Babbitt alloys, studies on the preparation of Babbitt alloy coatings using annular laser coaxial wire-feed cladding technology remain relatively limited. This study systematically compares the microstructural characteristics and mechanical properties of Babbitt alloys prepared by conventional casting and annular laser coaxial wire-feed cladding processes, aiming to elucidate the influence mechanisms of annular laser cladding on the microstructural evolution and mechanical properties of Babbitt alloys, thereby providing theoretical foundation and technical support for the development of novel high-performance bearing alloy layers.

## 2. Experiment Method and Devices

### 2.1. Laser Cladding of Babbitt Alloy Layer on Bearing Steel Substrate

**Figure 1:**
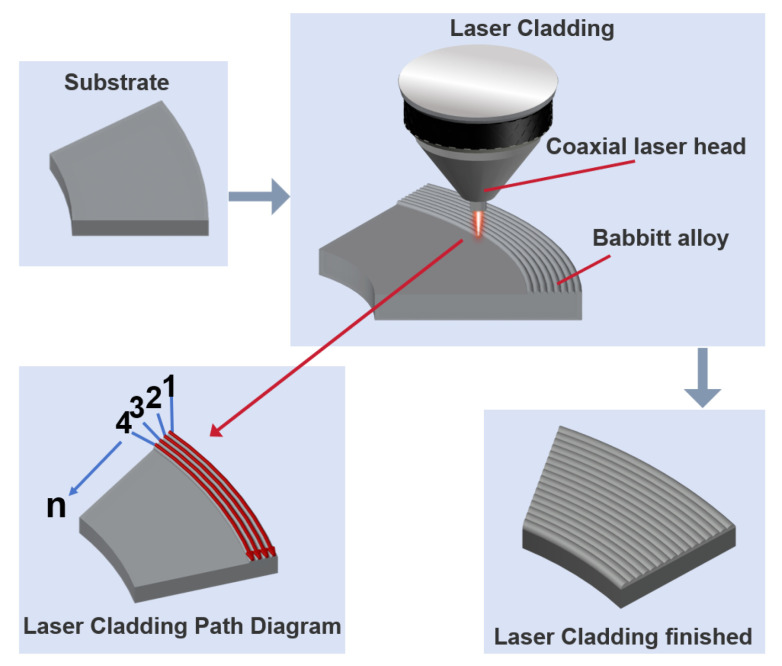
Schematic Diagram of Annular Laser Cladding Process.

Grade 25 steel (GB/T 699-2015) [25] was selected as the substrate material, and tin-based Babbitt alloy ZSnSb_11_Cu_6_ wire with a diameter of 1.6 mm was employed as the cladding material. The laser cladding system primarily consisted of a Raycus Rfl-C3000S fiber laser and a Tolertek TL-WP-F100-F160-A-OTP coaxial wire-feeding laser head, both provided by Shanghai Tongli Laser Technology Co., Ltd. The experimental procedure comprised four main steps (Figure 1). (1) Steel surface pretreatment. The steel substrate surface was mechanically ground and subsequently subjected to ultrasonic cleaning in anhydrous ethanol, followed by surface drying. These procedures effectively eliminated surface impurities, corrosion products, and oxidation films, thereby ensuring interfacial cleanliness essential for the formation of superior metallurgical bonds. (2) Parameter setting and cladding path planning. Based on preliminary process optimization, the laser power was set to 2400 W, the wire feeding speed to 2.8 m min^−1^, the overlap offset to 1.7 mm, and the traverse speed to 25 mm s^−1^. The cladding path is designed using a unidirectional continuous scanning approach, beginning at one side of the substrate and ending at the other side, as illustrated in Figure 1. This strategy is implemented to avoid the temperature field asymmetry and molten pool turbulence phenomena commonly caused by bidirectional scanning. (3) Laser cladding process. Upon initiation of the laser cladding system, the wire material rapidly melts under the high-energy density laser (providing a linear energy input of 96 kJ/m and an energy density of approximately 43.6 J/mm^2^ over the annular spot), forming molten droplets that are continuously deposited. Concurrently, the laser energy partially melts the surface layer of the steel substrate, allowing the molten Babbitt alloy to thoroughly mix with the liquid steel substrate, thereby forming a metallurgical bonding interface. As the laser beam moves, the molten pool rapidly solidifies, resulting in a dense and uniform Babbitt alloy cladding layer. (4) Cooling and post-treatment. Following the completion of the cladding process, the deposited layer was naturally cooled to ambient temperature, which served to minimize residual stresses and mitigate crack initiation. Subsequently, upon complete cooling, post-treatment procedures comprising grinding and polishing were conducted to eliminate surplus alloy material and surface oxidation.

### 2.2. Casting of Babbitt Alloy Layer on Bearing Steel Substrate

The static casting process for depositing tin-based Babbitt alloy ZSnSb_11_Cu_6_ on 25# steel involves the following steps: First, the steel substrate is pre-treated using the same method as in laser cladding, and a solution of hydrochloric acid and ammonium chloride is applied to ensure the quality of the cast surface. Next, the cleaned mold is placed in a heating furnace and preheated to 150–200 °C to ensure uniform heating and prevent cracks due to temperature differences. The Babbitt alloy raw material is then heated in a furnace to 400–450 °C, with continuous stirring to ensure uniform composition and the removal of any surface dross. Once the alloy is fully melted and at the correct temperature, it is slowly poured into the preheated mold, maintaining a steady flow to avoid splashing and the formation of air bubbles. The casting is allowed to cool naturally to room temperature within the mold before demolding. Finally, the casting is cleaned and inspected, and any excess burrs and sprues are removed from the surface.

### 2.3. Structural and Performance Characterization

The surface morphology of the samples was examined using a JSM-6490LV scanning electron microscope (SEM, manufactured by JEOL Ltd., Tokyo, Japan). The SEM observations were conducted at an accelerating voltage of 15 kV. Phase composition was analyzed by X-ray diffraction (XRD, Bruker D8, Bruker Corp., Karlsruhe, Germany). The measurements were conducted using Cu Kα radiation (λ  =  0.154 nm) over a 2θ range of 25–90° with a step size of 0.02°. Phase identification was carried out based on the PDF-2 2004 database without background subtraction. Metallographic observations were conducted with an OLYMPUS DSX1000 ultra-depth 3D digital microscope (manufactured by Olympus Corp., Tokyo, Japan). The polished samples were etched with a 4% nitric acid alcohol solution, thoroughly rinsed with deionized water, dried, and then examined under the microscope. The hardness of the Babbitt alloy layer was measured using a Laijin HBS-3000J digital Brinell hardness tester (manufactured by Laizhou Testing Machine & Diamond Tool Factory, Laizhou, China). A tungsten carbide (WC) spherical indenter with a diameter of 5 mm was employed. The tests were conducted under a load of 62.5 kgf with a dwell time of 60 s. Prior to hardness measurement, the test surfaces were prepared to a surface roughness of ≤1.6 µm (Ra) to minimize measurement deviations. Five individual measurements were taken at well-spaced locations on each sample to avoid any influence from adjacent indentations, and the average value was calculated as the reported hardness. The interfacial bond strength was determined by tensile testing in accordance with the Chinese National Standard GB GB/T 18329.2 [26] (equivalent to International Standard ISO 4386-2 [27]). The tests were performed using a WAW-300B microcomputer-controlled electro-hydraulic servo universal testing machine manufactured by Jinan Xinguang Testing Machine Manufacturing Co., Ltd. (Jinan, China). The geometry of the tensile specimen is shown in Figure S1 of the Supplementary Material, loading was applied at a constant displacement rate until failure occurred. Tribological properties were evaluated using an MFT-5000 multifunctional tribometer (Rtec Instruments, San Jose, CA, USA) in a reciprocating ball-on-disk configuration. A G10-grade precision steel ball with a diameter of 6.35 mm served as the counterpart. All tests were conducted at room temperature in ambient air under dry sliding conditions without lubrication. The specific test parameters were as follows: a normal load of 5 N, a reciprocating stroke of 5 mm, a frequency of 1 Hz, and a total test duration of 3600 s (corresponding to 3600 cycles). The coefficient of friction was recorded in real time by the instrument’s software. One test was performed per sample. The wear volume was quantified using three-dimensional profilometry, and the specific wear rate was subsequently calculated using the provided formula. The formula for specific wear rate (1) is:

(1) $$K=\frac{∆V}{Fn\times L}$$

where ∆*V* is the wear volume, *Fn* is the normal load, and *L* is the total sliding distance.

## 3. Results and Discussion

### 3.1. Surface Quality and Miacro-Structure of the Alloy Layer

**Figure 2:**
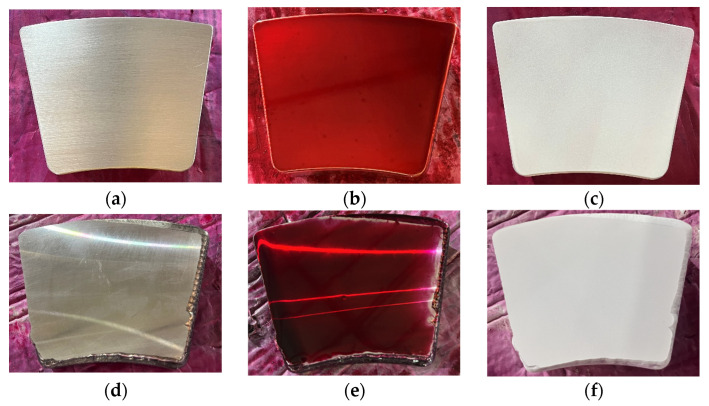
Dye penetrant inspection of the alloy layers. (**a**) Surface of the cast Babbitt alloy. (**b**) Surface coloring of cast alloy layer. (**c**) the cast Babbitt alloy coated with penetrant developer. (**d**) Surface of the laser-clad Babbitt alloy. (**e**) Surface coloring of the laser-clad Babbitt alloy. (**f**) The laser-clad Babbitt alloy coated with penetrant developer.

The surface defects of the materials were examined using dye penetrant inspection (DPI). As shown in Figure 2, microscopic porosity was observed on the cast alloy surface, whereas the laser-clad alloy layer exhibited a uniform and dense microstructure with no significant penetrant residue or defect indications. These results further confirm the stability and reliability of the laser cladding process, demonstrating that optimized process parameters—including laser power, scanning speed, and powder feed rate—effectively mitigate surface cracks and porosity caused by thermal stress concentration or excessive cooling rates during the cladding process. Additionally, the superior surface quality of the cladding layer indicates that the Babbitt alloy powder achieved complete melting and uniform distribution within the molten pool, thereby ensuring the structural integrity and continuity of the cladding layer.

### 3.2. Microstructural Analysis by Metallographic Examination

Microstructural characterization of the alloy layer was performed through metallographic analysis. According to the Sn-Sb-Cu ternary phase diagram [28], when the copper content (mass fraction) ranges from 0.5% to 8% and the antimony content exceeds 8%, the microstructure of the Babbitt alloy primarily consists of three phases: the Sn solid solution phase, the SnSb and the Cu_6_Sn_5_ intermetallic compound [29,30].

**Figure 3:**
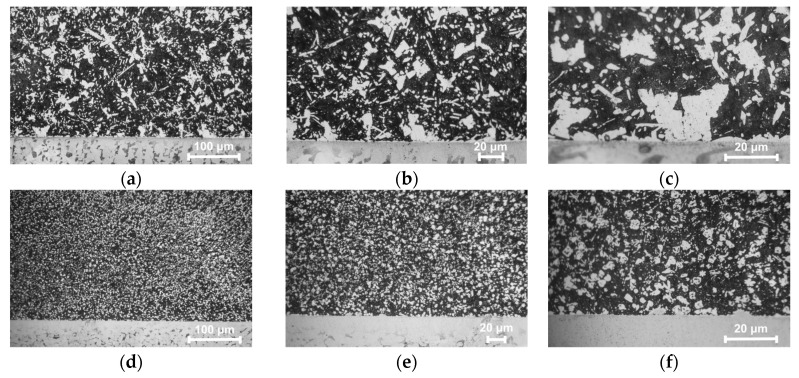
Metallographic Image of the Alloy Interface. (**a**–**c**) Cast sample. (**d**–**f**) Laser clad sample.

To characterize the interfacial microstructure, metallographic analysis was performed. As shown in Figure 3, micrographs of the interface for both samples are presented at magnifications of 100×, 200×, and 500×. In all micrographs, the Babbitt alloy is located above the substrate. In the as-cast samples (Figure 3a–c), the microstructure exhibits coarse SnSb phases with blocky and dendritic morphologies embedded in the soft black Sn matrix, along with finely dispersed Cu_6_Sn_5_ phases. The SnSb grains are notably large, reaching up to ~100 μm in size, and exhibit interconnected grain structures. In contrast, the laser-clad samples (Figure 3d–f) demonstrate a significantly refined microstructure, where the SnSb phase grain size is reduced to ~10–20 μm, and both the SnSb and Cu_6_Sn_5_ phases are uniformly distributed [31,32].

**Figure 4:**
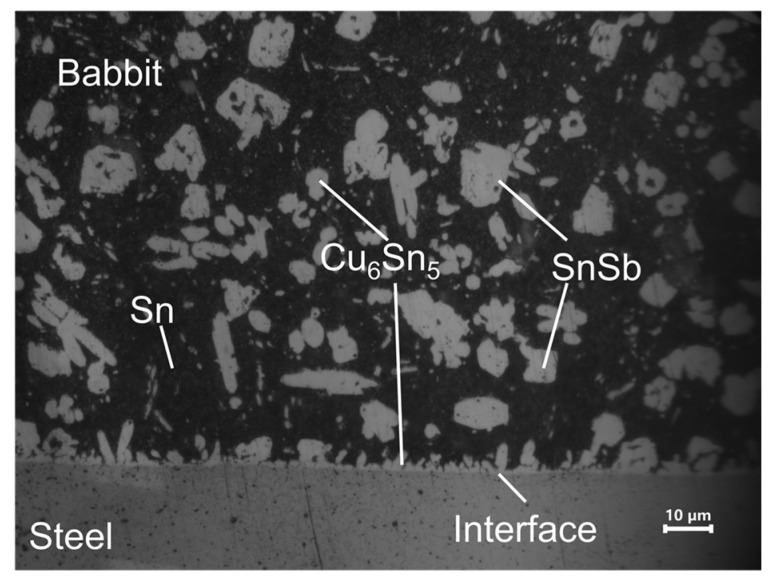
Metallographic image of the laser-clad alloy interface under high magnification.

High-magnification metallographic observation (Figure 4) reveals that the Babbitt alloy grains in the laser-clad samples exhibit preferred growth along the interface, confirming a well-established metallurgical bond. Notably, the interfacial region is predominantly enriched with the Cu_6_Sn_5_ phase, which effectively impedes crack propagation and enhances interfacial bonding strength. Additionally, the fine and homogeneous distribution of the SnSb phase contributes to improved fatigue resistance [33]. These results demonstrate that, under the experimental conditions of this study, the laser cladding process can achieve satisfactory interfacial bonding without preheating the steel substrate, providing a basis for process simplification.

### 3.3. Crystal Structure and Phase Analysis of the Alloy Layer

**Figure 5:**
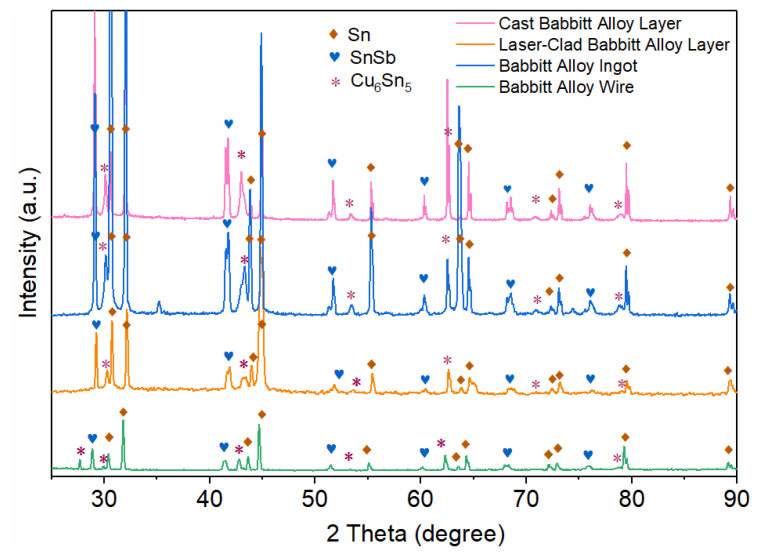
XRD patterns of the Cast Babbitt alloy layer, laser-clad Babbit alloy layer, Babbit alloy ingot and the Babbit alloy wire.

To investigate the influence of different preparation processes on the phase composition of babbitt alloy, X-ray diffraction (XRD) analysis was performed on four samples: the Babbitt alloy ingot, the cast alloy layer, the Babbitt alloy wire, and the laser-clad Babbitt alloy layer (Figure 5).

The results reveal that all samples consist of the Sn matrix phase (PDF No. 04-0673) [34,35,36], the SnSb intermetallic compound phase (PDF No. 33-0118) [37], and the Cu_6_Sn_5_ intermetallic compound phase (PDF No. 45-1488) [38]. Quantitative analysis indicates that the mass fractions of the Sn phase in Babbitt alloy ingot, as-cast Babbitt alloy layer, Babbitt alloy wire and laser-clad Babbitt alloy layer are 69.45%, 58.36%, 40.51% and 55.61%, respectively; those of the SnSb phase are 14.49%, 21.57%, 13.96% and 14.75%; while the Cu_6_Sn_5_ phase, which serves as the main strengthening phase, accounts for 16.06%, 20.07%, 45.53% and 29.64% in the corresponding samples.

Notably, the strongest diffraction peak of the Sn phase in the laser-clad Babbitt alloy layer appears at 2θ = 44.9°, whereas the corresponding strong peak of the Sn phase in the cladding wire is located at 2θ ≈ 32.0°. This peak shift may originate from a preferred orientation or residual stress induced by the extremely high cooling rate during the laser-cladding process [39]. In contrast, the diffraction peak positions of the SnSb and Cu_6_Sn_5_ phases remain highly stable across all samples. Their strongest peaks are located at 2θ ≈ 29.0° (corresponding to the (002) plane) and 62.6°, respectively, consistent with the standard PDF cards, confirming the good stability of their crystal structures. The higher content of the hard Cu_6_Sn_5_ phase in the laser-cladded alloy layer contributes to the enhancement of its hardness, which is in good agreement with the hardness test results presented later in this work.

### 3.4. Microstructure Morphology Analysis of the Alloy Layers

**Figure 6:**
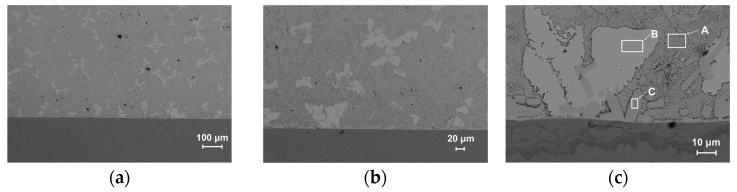

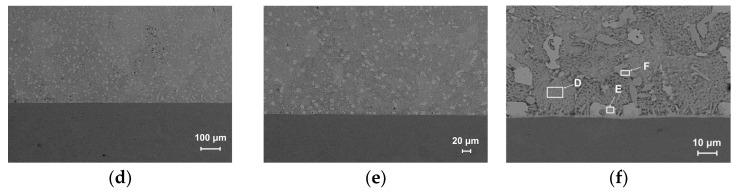
SEM of samples prepared by two methods. (**a**–**c**) Cast alloy layer microstructure at 100×, 200×, and 1000× magnification. (**d**–**f**) Laser clad alloy layer microstructure at 100×, 200×, and 1000× magnification. Regions A and D correspond to the Sn phase, regions B and E represent the SnSb phase, and regions C and F correspond to the Cu_6_Sn_5_ phase.

To comprehensively investigate the surface micro-morphology at the interface between cast and laser-clad Babbitt alloy layers, SEM images of the etched interface were captured at various magnifications. Figure 6a–c and d–f illustrate the cross-sectional micro-morphology of the cast and laser-clad alloy layers at magnifications of 100, 200, and 1000 times, respectively. Consistent with the metallographic images, a bright blocky SnSb phase is embedded in the gray Sn substrate, accompanied by acicular Cu_6_Sn_5_. The laser-clad layer exhibits finer grains and is free of pores and cracks, with a uniform distribution of constituents, indicating good densification of the alloy layer. Overall, the laser-clad alloy layer displays homogeneous physical and chemical properties, which can prevent performance fluctuations caused by localized defects.

EDS compositional analysis of the phases in Figure 6c,f, summarized in Table 1, accurately quantified the chemical compositions of the phases in the as-cast (Figure 6c) and laser-clad (Figure 6d) samples, revealing three main phases: a Sn-rich matrix phase (β-Sn), a hard SnSb intermetallic, and a strengthening Cu_6_Sn_5_ intermetallic.

Regions A and D correspond to the Sn-rich matrix (β-Sn). The notably higher Sn content (77.59 wt%) and lower Sb content in the laser-clad layer (Region D) indicate that rapid solidification suppressed the solid solution of Sb in β-Sn, resulting in a purer matrix. In theory, a purer and softer Sn matrix facilitates the formation of a continuous lubricating film during sliding, which is expected to improve friction compatibility. Regions B and E are identified as the hard SnSb phase. The composition of the SnSb phase in the laser-clad layer (Region E: Sn 56.02 wt%, Sb 43.48 wt%) is closer to its theoretical stoichiometry (SnSb), and metallographic observations show a significantly refined microstructure. Such a nearly stoichiometric, fine and uniformly dispersed hard phase can act as an effective wear-resistant skeleton, distributing the load more uniformly and thus potentially enhancing the macro-scale wear resistance of the material. Regions C and F are both confirmed as the Cu_6_Sn_5_ phase. Their highly consistent compositions demonstrate that this phase’s crystal structure is stable regardless of the fabrication process. In the laser-clad layer, Cu_6_Sn_5_ is enriched at the interface, which contributes to strong metallurgical bonding, while its uniform distribution throughout the clad layer also provides overall strengthening.

In summary, laser cladding, through rapid solidification, reconstructs the microstructure: it promotes the precipitation of Sb to form fine, dispersed SnSb hard particles, while simultaneously distributing Cu uniformly to create a Cu_6_Sn_5_ strengthening network that is homogeneous overall and locally enriched at the interface. This synergistic structure—characterized by matrix purification, fine and dispersed hard phases, and a strengthening phase with dual-mode distribution (bulk-uniform plus interface-enriched) forms the microstructural basis for the improved material performance.

### 3.5. Hardness Measurement Results

**Figure 7:**
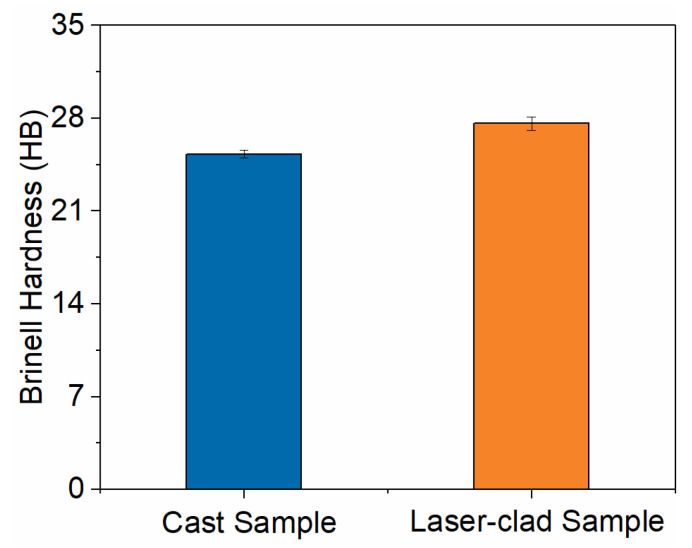
The Brinell hardness values of samples prepared by two methods.

To evaluate the hardness of the alloy layers, Brinell hardness tests were conducted using a digital hardness tester with a 5 mm diameter tungsten carbide indenter, under a test load of 62.5 kgf, and a dwell time of 60 s. Five indentations were measured, and the arithmetic mean was calculated. As shown in Figure 7, the average hardness of the cast Babbitt layer was 25.3 HB, while the average hardness of the laser-clad Babbitt layer was 27.6 HB, representing an increase of approximately 2.3 HB in the laser-clad layer compared to the cast layer. Metallographic and SEM analyses revealed significant grain refinement in the laser-clad layer, which can directly contribute to the hardness increase through the Hall–Petch effect.

### 3.6. Analysis of the Bonding Performance Between the Alloy Layer and Substrate

**Figure 8:**
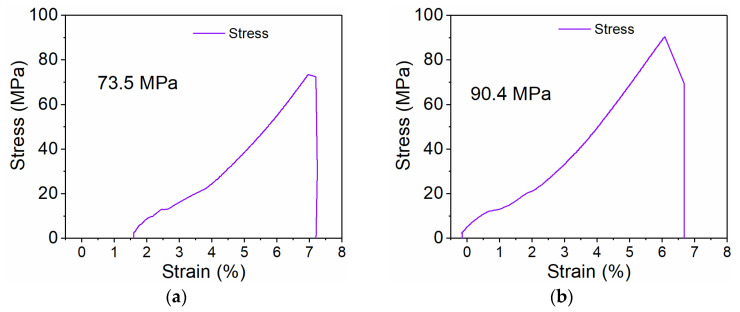
The Stress–Strain Curve Diagram. (**a**) The Stress–Strain Curve of cast sample. (**b**) The Stress–Strain Curve of laser-clad sample.

The interfacial bond strength between the Babbitt alloy layer and the steel substrate was determined by tensile testing. Figure 8 presents representative Stress–Strain Curves for the cast alloy layer and laser-clad alloy layer samples. The calculated bond strength of the cast specimen was 73.5 MPa, whereas that of the laser-clad sample was 90.4 MPa, corresponding to an increase of approximately 23.0% under identical test conditions. This marked improvement indicates that the laser-cladding process enhances interfacial bonding quality and strength. Microstructural analysis shows that the laser-clad layer has finer, denser grains, is free of pores and cracks, and exhibits a uniform elemental/phase distribution, all of which favor the formation of a robust metallurgical bond, reduce the risk of interface-related failure, and contribute to more stable in-service performance.

### 3.7. Tribological Performance of the Alloy Layer

**Figure 9:**
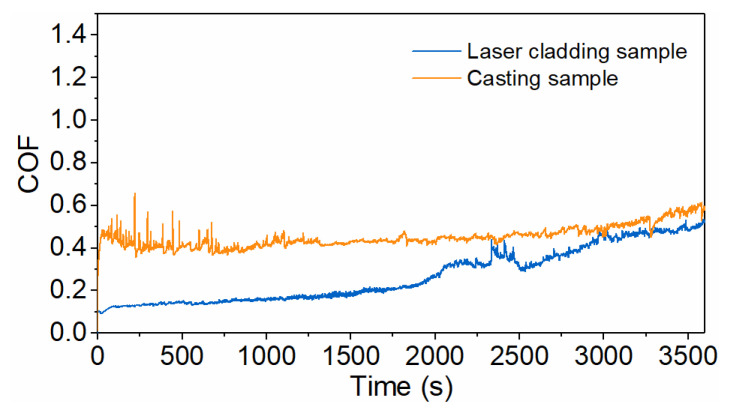
Friction coefficient curves of samples produced by laser cladding and casting processes.

Reciprocating ball-on-disk wear tests were performed to characterize the tribological behavior of the alloy layer. A G10-grade precision steel ball (diameter 6.35 mm) was used as the counterbody. Test conditions were set as follows: normal load 5 N, reciprocating stroke 5 mm, frequency 1 Hz, and test duration 3600 s. The coefficient of friction (COF) was recorded dynamically, and wear volumes was quantified by three-dimensional profilometry. Figure 9 presents the friction curves, where the blue line corresponds to the laser-clad sample and the yellow line to the cast sample. The average COF values were 0.4520 for the cast sample and 0.2794 for the laser-clad sample, i.e., the average COF of the laser-clad sample was reduced by approximately 38.19% relative to the cast sample.

The friction-coefficient-versus-time curves indicate that the as-cast sample exhibits large fluctuations in COF during the early stage of the test, and shows a gradual increase after approximately 3300 s. By contrast, the laser-cladded sample displays a lower overall COF, with an initial value of about 0.0926 that increases slowly at first, then the rate of increase accelerates after roughly 2000 s, and the COF approaches a steady state after approximately 3000 s.

**Figure 10:**
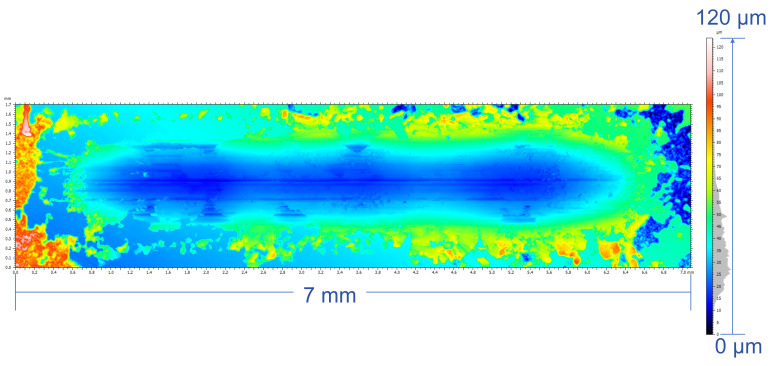
Friction and wear morphology of cast sample.

**Figure 11:**
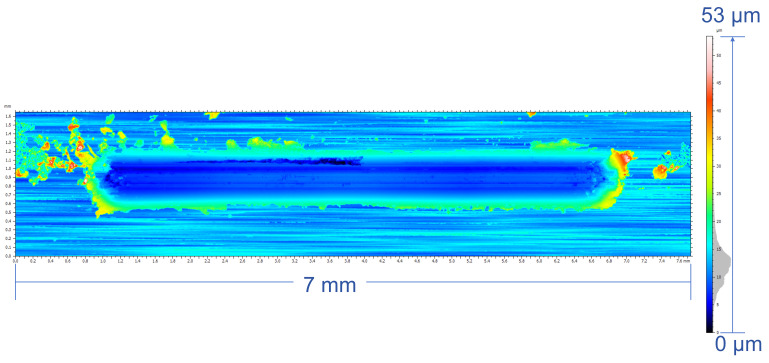
Friction and wear morphology of Laser-clad sample.

The pseudocolor wear maps and SEM morphology after the friction tests were presented in Figure 10 and Figure 11, revealing markedly different wear morphologies between the two samples. The wear scar of the as-cast alloy layer exhibits a characteristic three-lobed bulging morphology, with raised regions at both ends and a slightly narrower central bulge. This non-uniform wear pattern is consistent with a saddle-shaped contact stress distribution, in which the contact stress is highest at the ends and lower at the center. The observations indicate that the as-cast alloy layer exhibits a combined adhesive-abrasive wear mechanism, with localized fatigue spalling pits at coarse grain boundaries or porosity-induced defects, resulting in non-uniform wear width accompanied by significant material transfer and spalling [40,41].

**Figure 12:**
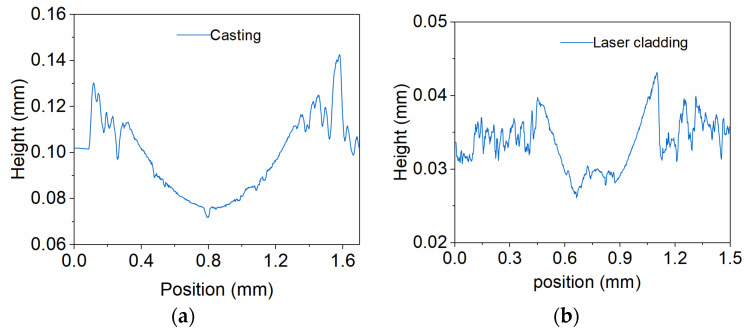
The 2D wear morphology cross-sectional profile. (**a**) Cast sample. (**b**) Laser-clad sample.

The 2D cross-sectional wear profile of the cast sample (Figure 12a) exhibits a wide groove, further corroborating this observation. In contrast, the laser-clad alloy layer showed predominantly mild abrasive wear, the surface exhibited regular, parallel frictional striations and no significant material transfer [42], features characteristic of abrasive wear under elastic contact conditions. These observations demonstrate that the laser-clad layer has higher hardness and a denser microstructure; grain refinement is beneficial for suppressing crack initiation, and fatigue spallation was not evident [36,43]. The cross-sectional wear profile of the laser-clad sample (Figure 12b) exhibits a pronounced, deep V-shaped groove, consistent with abrasive wear being the predominant mechanism. These results are consistent with the metallographic, SEM and microhardness test conclusions. The wear volume of the laser-clad alloy layer was 4.78 × 10^−2^ mm^3^ with a maximum depth of 2.98 × 10^−2^ mm, while the wear volume of the cast alloy layer was 5.28 × 10^−2^ mm^3^ with a maximum depth of 3.54 × 10^−2^ mm. Consistent with the friction measurements, higher friction coefficients are associated with increased material weight loss [40,44].

The wear volume of the as-cast sample was approximately 10.46% greater than that of the laser-clad sample. The standardized volume wear rates of the laser-clad and as-cast alloy layers were 2.66 × 10^−4^ and 2.93 × 10^−4^ mm^3^·N^−1^·m^−1^, respectively. Overall, under identical test conditions, laser cladding markedly reduced the friction coefficient and improved wear resistance; this improvement is attributed to the finer, denser microstructure and higher hardness of the laser-clad layer, which act to suppress adhesive wear and fatigue spallation. These interpretations are supported by metallographic, SEM and microhardness analyses [45].

**Figure 13:**
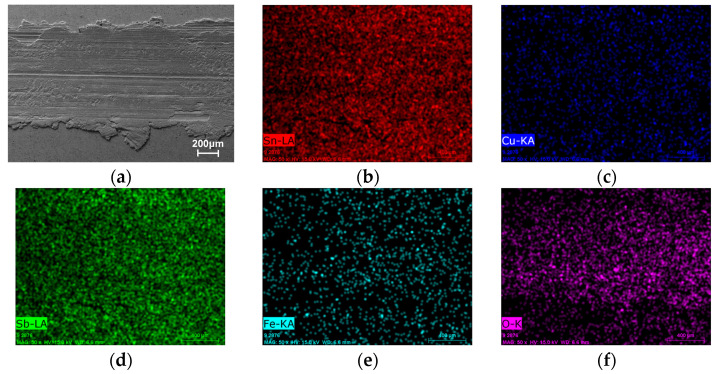
SEM image and corresponding EDS elemental maps of the wear track on the as-cast alloy layer. (**a**) SEM of the wear track. (**b**–**f**) Distribution maps of Sn, Cu, Sb, and O, respectively.

**Figure 14:**
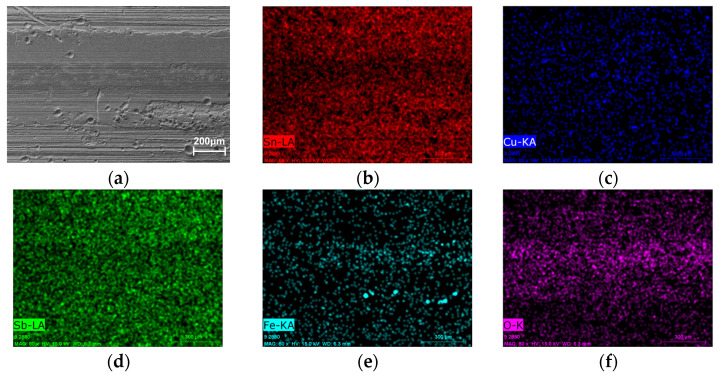
SEM image and corresponding EDS elemental maps of the wear track on the laser-clad alloy layer. (**a**) SEM of the wear track. (**b**–**f**) Distribution maps of Sn, Cu, Sb, and O, respectively.

**Figure 15:**
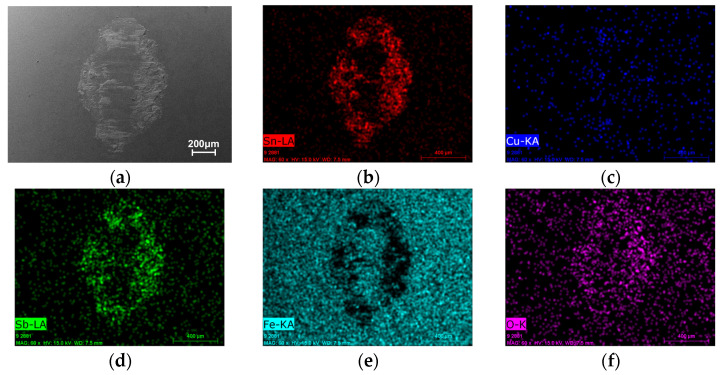
SEM image and corresponding EDS elemental maps of the wear track wear scar on the G10 steel ball counterpart after sliding against the as-cast Babbitt alloy layer. (**a**) SEM of the wear track. (**b**–**f**) Distribution maps of Sn, Cu, Sb, and O, respectively.

**Figure 16:**
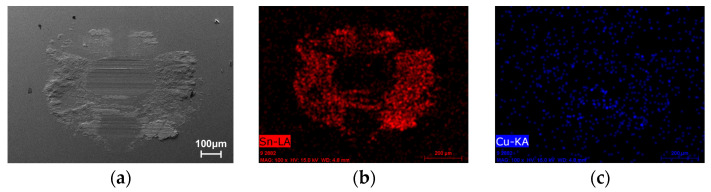

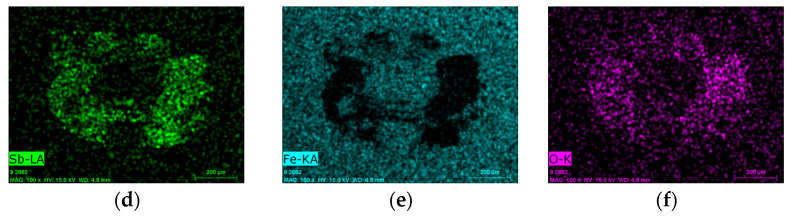
SEM image and corresponding EDS elemental maps of the wear track wear scar on the G10 steel ball counterpart after sliding against the laser-clad Babbitt alloy layer. (**a**) SEM of the wear track. (**b**–**f**) Distribution maps of Sn, Cu, Sb, and O, respectively.

To further investigate the wear mechanisms, EDS elemental mapping was performed on the wear track surfaces (Figure 13, Figure 14, Figure 15 and Figure 16). Signals corresponding to Fe and O were detected in the wear tracks of both the as-cast and laser-clad alloy layers; however, their distribution patterns and relative intensities revealed fundamentally different dominant wear mechanisms.

In the cast alloy wear track (Figure 13), the Fe signal within the wear track was strong and uniformly distributed. Combined with the SEM observation of severe material extrusion and smearing, this indicates that extensive, large-scale adhesive transfer occurred at an early stage of sliding. Material from the counterpart steel ball was continuously smeared onto the relatively soft as-cast surface. This is further corroborated by the distinct chemical signature on the counterpart ball itself, where its wear scar (Figure 15) shows a clear enrichment of Sn, Cu, and Sb elements transferred from the Babbitt alloy. The concurrent uniform distribution of O suggests that oxidation accompanied this process. The resulting composite structure—a soft matrix overlaid with a hard transferred layer- is prone to instability and spallation under cyclic stressing, generating third-body abrasives that intensify three-body abrasion, ultimately leading to a progressively increasing and highly fluctuating coefficient of friction.

In contrast, for the laser-clad alloy wear track (Figure 14), the overall Fe signal in the wear track was significantly weaker and exhibited pronounced local reduction. Meanwhile, clear enrichment of Sn, Cu, and Sb elements was detected at the periphery of the wear scar on the counterpart steel ball (Figure 16). This evidence demonstrates that a limited and controlled material exchange took place at the clad surface. Due to the higher hardness of the clad layer, adhesive effects were initially suppressed. As sliding progressed, trace amounts of surface material (Sn, Sb, Cu) underwent mild transfer to the steel ball under frictional heating and pressure, forming an ultrathin, sacrificial transfer film. This film, in turn, protected the underlying clad layer, thereby preventing extensive reverse transfer of Fe from the steel ball. The distribution of O within the wear track indicates that slight oxidation accompanied this process.

These mechanistic interpretations align perfectly with the macro-scale performance: the as-cast alloy, unable to resist severe adhesive transfer, entered a detrimental cycle of “material loss–oxidation–spallation,” manifesting as a high, fluctuating friction coefficient and large wear volume. In contrast, the laser-clad layer, owing to its higher hardness and refined microstructure, regulated the wear process into a stable regime dominated by mild abrasive wear, supplemented by controlled minor material exchange and oxidation. This resulted in a shallow, regular V-shaped wear track, a stable and low friction coefficient, and a significantly reduced wear volume. Consequently, the superiority of the laser-cladding process lies not only in enhancing the intrinsic material properties but also in actively guiding and stabilizing the dynamic behavior of the entire tribo-system.

## 4. Conclusions

Based on a systematic comparison of microstructure, mechanical properties, and tribological performance, annular laser coaxial wire-feed cladding demonstrates clear advantages over conventional casting for preparing Babbitt alloy layers. The laser-clad layer exhibits a uniform, defect-free surface and a significantly refined microstructure, with the SnSb phase grain size reduced from ∼100 μm (as-cast) to ∼10–20 μm. A sound metallurgical bond characterized by interfacial enrichment of the Cu_6_Sn_5_ strengthening phase is formed at the interface. The tensile bond strength reaches 90.4 MPa for the laser-clad layer—approximately 23.0% higher than that of the as-cast layer (73.5 MPa). The macro-hardness of the laser-clad layer is 27.6 HB, about 9% greater than that of the as-cast counterpart (25.3 HB). In tribological tests, the average coefficient of friction of the laser-clad layer is reduced to 0.2794 (∼38% lower than the 0.4520 of the as-cast layer), and its wear volume is decreased by about 10.5% (4.78 × 10^−2^ mm^3^ vs. 5.28 × 10^−2^ mm^3^). These findings collectively confirm that annular laser cladding effectively refines the microstructure, strengthens interfacial bonding, and enhances both mechanical and wear-resistant properties, offering a promising technical route for fabricating high-performance bearing surfaces. This study primarily focused on comparing the macroscopic properties of the alloy layers. Due to the scope of the work, a quantitative characterization of the gradient in micromechanical properties (e.g., nano-hardness) across the steel/alloy interfacial transition zone was not performed. Future research will employ advanced characterization techniques such as nanoindentation grid mapping and micropillar compression to establish quantitative relationships between the interfacial microstructure and local mechanical properties.

## Figures and Tables

**Table 1:** Results of the EDS analysis conducted on the Phases observed in Figure 6c,d.

EDS Scanned Region	Possible Phases	Compositional Elements (wt.%)		
		Sn	Sb	Cu
A	Sn	69.32	28.01	2.66
B	SnSb	57	43	0.00
C	Cu_6_Sn_5_	58.89	4.06	37.05
D	Sn	77.59	22.41	0.00
E	SnSb	56.02	43.48	0.51
F	Cu_6_Sn_5_	58.60	5.24	36.16

## Data Availability

The original contributions presented in this study are included in the article/Supplementary Material. Further inquiries can be directed to the corresponding authors.

## Supplementary Materials

The following supporting information can be downloaded at https://www.mdpi.com/article/10.3390/mi17010134/s1. Figure S1. Schematic diagram of the tensile specimen geometry; Figure S2. Schematic illustration of the ball-on-disk reciprocating wear test setup.

## Abbreviations

The following abbreviations are used in this manuscript:

SEM	Scanning Electron Microscopy
EDS	Energy-Dispersive X-ray Spectroscopy
XRD	X-ray Diffraction
HB	Brinell Hardness
COF	Coefficient of Friction
LBID	Laser Beam Intensity Distribution
WIP	Workpiece Illumination Proportion
