# Supplementary material for: Microstructure and Properties of Conventional Cast Versus Annular Laser-Clad Babbitt Alloy Layers for Sliding Bearings
Source: Micromachines (Basel). 2026 Jan 21;17(1):134. doi: 10.3390/mi17010134 (PMC12844249; doi:10.3390/mi17010134)
Supplement: Supplementary file 1 [file micromachines-17-00134-s001.zip › micromachines-4005906-supplementary.pdf]

Microstructure and Properties of Conventional Cast versus Annular Laser-Clad Babbitt Alloy Layers for Sliding bearings

Supplementary Material

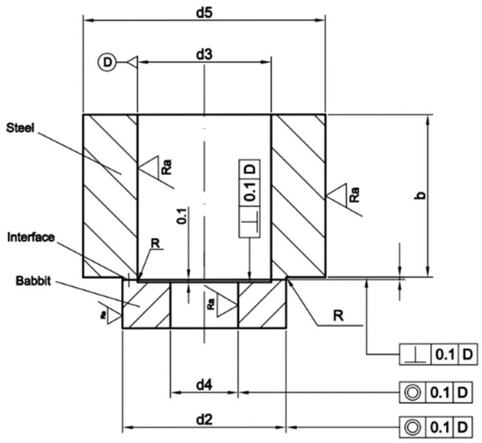

mm

d2 ±0.01	d3 ±0.01	d4 ±0.1	d5
19.58	16	8.1	29

Figure S1. Schematic diagram of the tensile specimen geometry.

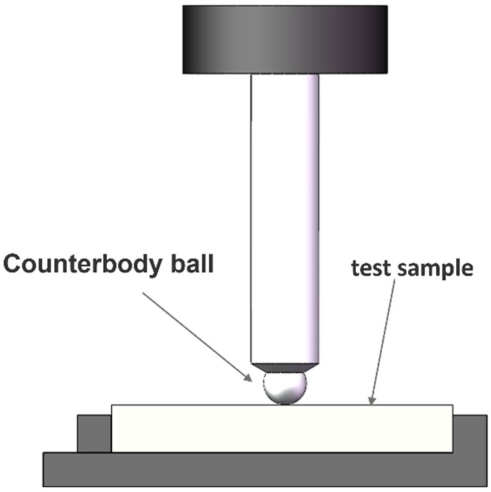

Figure S2. Schematic illustration of the ball-on-disk reciprocating wear test setup.
